# Urinary Exosomal MicroRNA Signatures in Nephrotic, Biopsy-Proven Diabetic Nephropathy

**DOI:** 10.3390/jcm9041220

**Published:** 2020-04-23

**Authors:** Wen-Chin Lee, Lung-Chih Li, Hwee-Yeong Ng, Pei-Ting Lin, Terry Ting-Yu Chiou, Wei-Hung Kuo, Chien-Te Lee

**Affiliations:** 1 Division of Nephrology, Department of Internal Medicine, Kaohsiung Chang Gung Memorial Hospital and Chang Gung University College of Medicine, Kaohsiung 83301, Taiwan; 2 Chung Shan Medical University School of Medicine, Taichung 40201, Taiwan

**Keywords:** microRNA, diabetic nephropathy, nephrotic

## Abstract

Diabetic kidney disease (DKD) is the leading cause of chronic kidney disease (CKD). Elucidating the mechanisms underlying proteinuria in DKD is crucial because it is a common problem in DKD-related mortality and morbidity. MicroRNAs (miRs) associated with DKD have been detected in experimental diabetes models and in patients with both diabetes and CKD. Here, we aimed to investigate pathologic miRs in diabetic nephropathy (DN) by prospectively following six nephrotic, biopsy-proven isolated DN patients (enrolled between August 2015 and July 2017) for one year. The urinary exosomes were isolated at the time of the biopsy and the contained miRs were analyzed by next-generation sequencing. The results were compared to the control group, composed of age-, gender-, and CKD stage-matched patients with proteinuric CKD who did not present diabetes. Among the 72 identified miRs, we investigated eight (miR-188-5p, miR-150-3p, miR-760, miR-3677-3p, miR-548ah-3p, miR-548p, miR-320e, and miR-23c) exhibiting the strongest upregulation (13–15 fold) and two (miR-133a-3p and miR-153-3p) with the strongest downregulation (7–9 fold). The functional analysis of these miRs showed that they were involved in known and novel pathways of DN, supporting their pathologic roles. The bioinformatics-based prediction of the target genes of these miRs will inspire future research on the mechanisms underlying DN pathogenesis.

## 1. Introduction

Diabetic kidney disease (DKD) has emerged as a leading cause of chronic kidney disease (CKD) worldwide. It occurs in approximately 30% and 40% of patients with type 1 and 2 diabetes mellitus, respectively [1,2,3], and is more common in low- and lower-middle-income countries [4]. The clinical diagnosis of DKD is based on the measurement of the estimated glomerular filtration rate (eGFR) and the albuminuria/proteinuria, along with clinical observations, including diabetes duration and the presence of diabetic retinopathy [5,6]. The major threats presented by DKD are risks of mortality, cardiovascular disease, end-stage renal disease, and infections, and a common risk factor for these DKD-related complications is proteinuria [7,8,9]. A better understanding of the mechanisms underlying proteinuria in DKD may help to develop novel treatment strategies for this aspect of DKD.

Nephrotic-range proteinuria is the most severe form of proteinuria in diabetic patients. It may result from isolated diabetic nephropathy (DN), non-diabetic renal disease, or combined nephropathies [10,11,12]. Renal biopsy is currently the only diagnostic tool able to delineate the renal pathologies in diabetic patients [12]. However, the invasiveness and cost of this technique, as well as the risk of tissue-sampling errors limit its general applicability to the diabetes patient population [13,14]. Non-invasive urine analysis is an appropriate approach to assess functional changes because urine contains proteins from the filtered plasma. However, only a few of these proteins are derived from the kidney, and structural changes in this organ are poorly detected through urine [15]. Urinary exosomes are normally secreted by cells from all nephron segments and mostly carry protein, mRNA, and microRNA (miR) markers of renal dysfunction and structural injury [16]. Urinary-exosomal miR analysis has emerged as a promising tool for the discovery of DKD biomarkers [15,17]. To date, the reported urinary-exosomal miR profiles for DKD are likely to be analyses based on a mixture of isolated DN, non-diabetic renal disease and combined nephropathies. In the current study, we examined the urinary-exosomal miR signatures of the nephrotic, biopsy-proven, isolated DN patients. 

## 2. Materials and Methods

### 2.1. Patients

Fifteen diabetic patients with nephrotic range proteinuria, who underwent a renal biopsy in our hospital between August 2015 and July 2017, were screened for this study. The diabetic patients with biopsy-proven non-diabetic renal disease or combined nephropathies were excluded. Other exclusion criteria included any febrile illness during the last three months, chronic inflammatory or rheumatic disease, hepatitis, HIV infection, and hereditary renal disease. The six patients fulfilling these criteria were prospectively followed at our nephrology outpatient clinic for one year. The control patients were non-diabetic CKD patients whose urinary protein-to-creatinine ratio (UPCR) ranged from 500 to 1000 mg/g. Blood samples and 20 mL of voided midstream urine were collected for routine laboratory testing. Due to the variability in the urinary protein excretion, two out of three specimens collected within a 3- to 6-month period should provide abnormal results before considering that a patient developed an increased UPCR. The urine samples were used immediately for exosomal miR separation. This study was approved by the Chang Gung Medical Foundation Institutional Review Board. All methods were performed in accordance with the relevant guidelines and regulations, and informed consent was obtained from all participants.

### 2.2. Isolation of Urinary-Exosomal RNA

Urinary-exosomal RNA was isolated using the exosome RNA isolation kit (Norgen-Biotek, Thorold, USA) according to the manufacturer’s protocol, as previously described [18]. Briefly, the RNA was extracted from 1 mL of urine. The RNA extractions were eluted in 100 μL and stored at −80 °C until use. The purified RNA was quantified at OD260nm by using a ND-1000 spectrophotometer (NanoDrop Technology, Wilmington, NC, USA) and was qualitated by using a Bioanalyzer 2100 (Agilent Technology, USA) with the RNA 6000 labchip kit (Agilent Technologies, San Jose, CA, USA). 

### 2.3. Library Preparation and Sequencing

The small RNA library construction and deep sequencing was commercially carried out at a biotechnology company (Welgene, Taipei, Taiwan). The samples were prepared using the Illumina sample preparation kit according to the TruSeq Small RNA Sample Preparation Guide. The 3′ and 5′ adaptors were ligated to the total RNA and reverse transcription, and followed by a polymerase chain reaction (PCR) amplification. The enriched cDNA constructs were size-fractionated and purified on a 6% polyacrylamide gel electrophoresis and on the bands containing the 18–40 nucleotide-RNA fragments (140–155 nucleotides in length with both adapters). The libraries were sequenced on an Illumina instrument (75SE cycle single-read). The sequencing data were processed with the Illumina software.

### 2.4. Small RNA- Sequencing Analysis

Initially, the sequences generated went through a filtering process to obtain the qualified reads. Trimmomatic [19] was implemented to trim or remove the reads according to the quality score. The qualified reads, after filtering low-quality data, were analyzed using miRDeep2 [20] to clip the 3’ adapter sequence and discard the reads shorter than 18 nucleotides, before aligning the reads to the human genome from University of California, Santa Cruz (UCSC). Only the reads that mapped perfectly to the genome five or fewer times were used for miR-detection, since miRs usually map to few genomic locations. MiRDeep2 estimates the expression levels of known miRs. The miRs with two-fold changes in expression and *p* value < 0.05 were selected for the target-genes prediction by using the miRmap database.

The output files were further annotated by adding gene-functional descriptions and gene ontology (GO) classifications. The reference genomes and gene annotations were retrieved from the Ensembl database (http://asia.ensembl.org/index.html). The GO term and fold enrichment, or the depletion for the gene lists of significantly up- and down-regulated genes in the urine exosomes, were determined. The GO analysis for significant genes was performed using the Kyoto Encyclopedia of Genes and Genomes (KEGG, http://www.genome.jp/kegg/) and the National Institutes of Health (NIH) Database for Annotation, Visualization and Integrated Discovery (DAVID) Bioinformatics Resources 6.7. 

## 3. Results

### 3.1. Patient Characteristics

According to the inclusion and exclusion criteria, six patients were prospectively followed at our nephrology outpatient clinic for a year. The clinical characteristics of the six study patients are summarized in Table 1. Their urine samples were collected for exosome isolation and analyzed by next-generation sequencing. At biopsy, Patient 4 was at Stage 1 CKD; meanwhile, Patients 1 and 2 were at Stage 4; and Patients 3, 5, and 6 were at Stage 3. All the patients had nephrotic range proteinuria. Their UPCR ranged from 5470.6 to 20364.4 mg/g. Four of the six patients had poor blood-sugar control. Their glycated- hemoglobin (HbA1C) levels were between 10.4% and 14.9%. All the study patients were on standard medications, including anti-diabetic agents, renin-angiotensin system (RAS) blockades, and statins. Patient 1 was on a low dose of methylprednisolone (2 mg/day). 

### 3.2. Urinary-Exosomal miR Signatures

**Figure 1:**
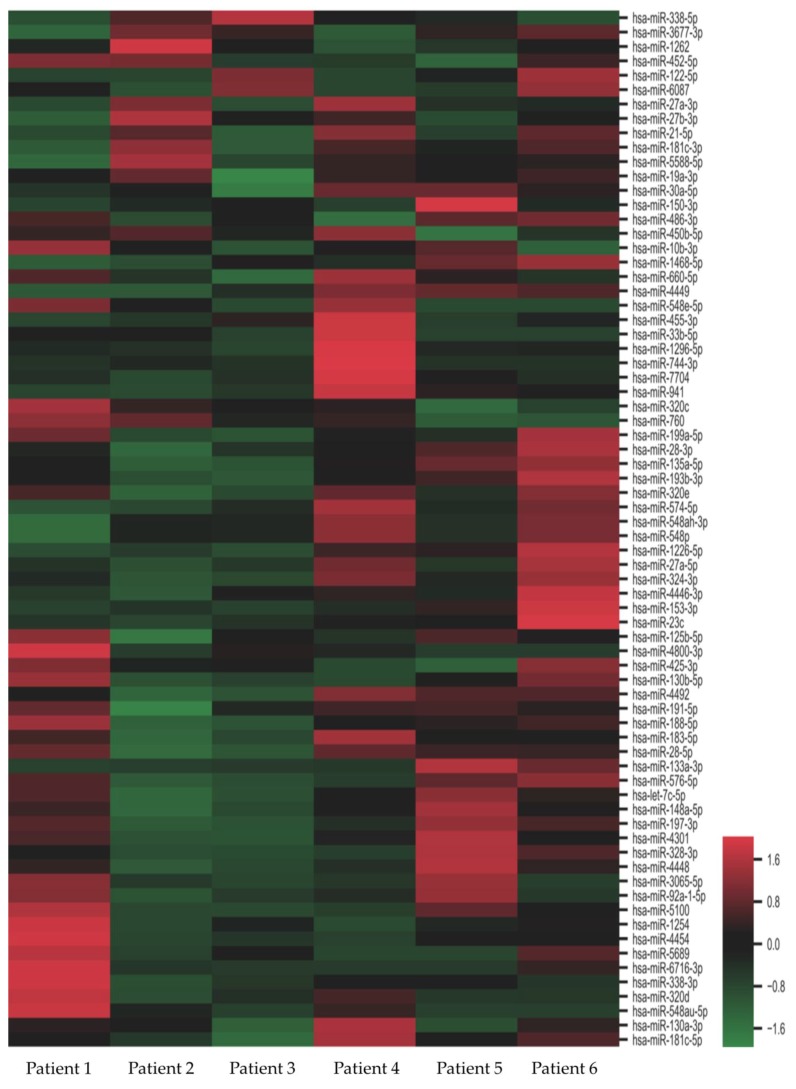
Heat map of differentially-expressed microRNAs (miRs) in nephrotic diabetic nephropathy (DN) patients. Fold change of expression levels were normalized to the mean signal intensities of the controls. Red and green colors represent fold change up- and down-regulation, respectively, as indicated by the linear scale bar.

**Figure 2:**
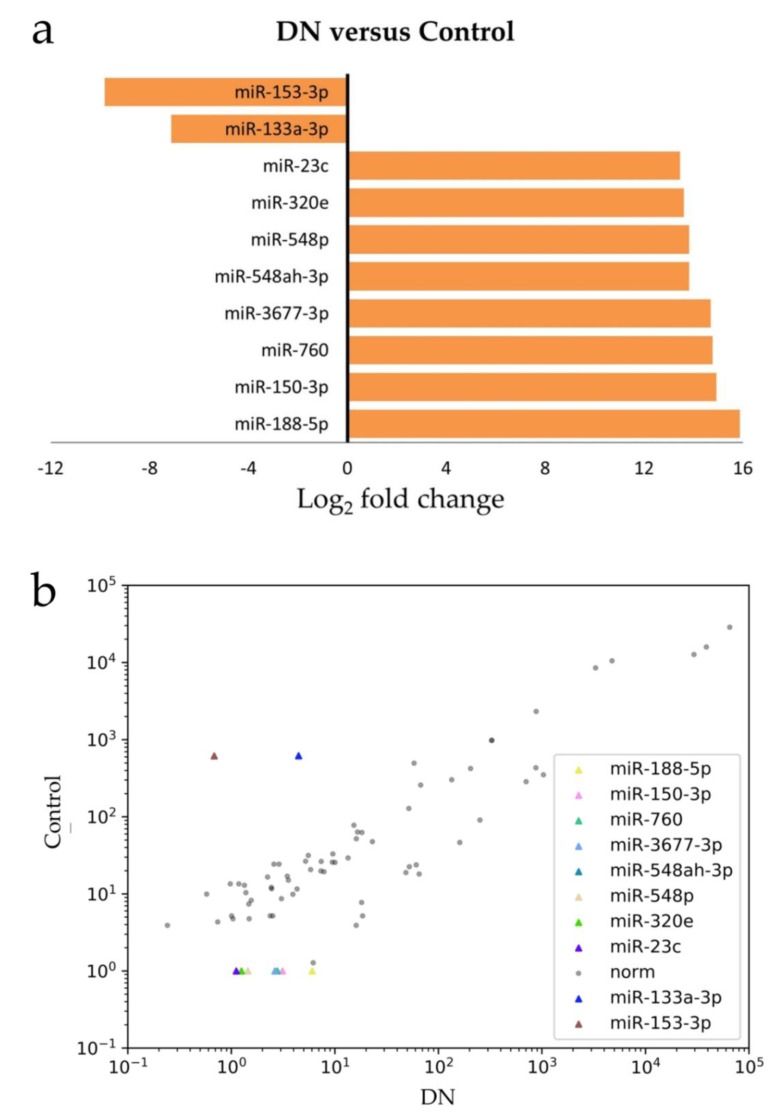
The most deregulated miRs in DN: (**a**) the differentially-expressed miRs in DN (versus control) are shown in Log_2_ fold change, with a positive value referring to up-regulation and a negative value meaning down-regulation; (**b**) the scatter plot shows that these deregulated miRs exhibited correlated-expression pattern between DN and control.

When comparing the next-generation sequencing data with age-, gender-, and CKD stage-matched controls, we identified 72 miRs showing significant differences in abundance (Figure 1). Among the 72 miRs identified, 22 were up-regulated and 50 were down-regulated. Eight miRs (miR-188-5p, miR-150-3p, miR-760, miR-3677-3p, miR-548ah-3p, miR-548p, miR-320e, and miR-23c) showed the strongest up-regulation (13–15 fold) and two (miR-133a-3p, miR-153-3p) showed the strongest down-regulation (7–9 folds). These deregulated miRs exhibited a correlated expression pattern between DN and control (Figure 2). The attributed functions of the deregulated miRs, based on published data, are summarized in Table 2.

The strongest up-regulation that we identified in the urine exosomes of the nephrotic, isolated DN patients was with miR-188-5p. This miR has been reported to regulate the high glucose-induced epithelial-mesenchymal transition of HK-2 cells through the PTEN/PI3K/Akt pathway [21]. The second most strongly up-regulated miR was miR-150-3p. It has been reported to be up-regulated in the circulating exosomes of clinically-diagnosed DN patients [22]. In addition, we described for the first time that miR-760, miR-3677-3p, miR-548ah-3p, miR-548p, miR-320e, and miR-23c were up-regulated in the DN patients. Although miR-133a-3p and miR-153-3p have been reported to have implications in pig kidneys [24] and lupus nephritis [25], this is the first time they were reported in the nephrotic DN patients.

### 3.3. Functional Analysis of the Identified miRs

Using the KEGG and NIH DAVID Bioinformatics Resources, we obtained the predicted pathways targeted by the miRs that were identified in nephrotic DN patients. The P-values of significance-over-background are shown alongside the results of the analysis (Table 3). These pathways included PI3K-Akt-signaling, mitogen-activated protein kinase (MAPK)-signaling, human papillomavirus infection, RAS-signaling, endocytosis, and pathways regulating the pluripotency of stem cells. In these pathways, the target molecules of the miRs we examined are summarized in Table 4. Many of the listed target genes are novel and not yet characterized in experimental diabetes models. In the PI3K-Akt-signaling pathway, miR-548p was predicted to target EIF4E, FGFR1 and PRLR. Meanwhile, miR-548ah-3p was involved in the PI3K-Akt-signaling pathway by targeting CCND1, PRKAA2 and PPP2R5E. In the MAPK-signaling pathway, the predicted-target genes included MAP3K7, FGFR1, MKNK2, TGFBR1, RPS6KA3, NF1, AKT3, STK4 and FGF1. The miR-23c, miR-548p, miR-548ah-3p, and miR-320e played roles in the human papillomavirus infection pathway through their target genes. By targeting different genes, miR-548ah-3p played roles in all these functional pathways. Except for the endocytosis pathway, an interesting finding was that miR-320e regulated AKT3, which was the hub of the other five pathways.

## 4. Discussion

Although various miRs have been reported to be related to diabetic nephropathy, these results need to be cautiously interpreted. Some studies have identified miRs in cultured kidney cells [26], whereas others have analyzed urinary-exosomal miRs from patients who have simultaneously suffered from diabetes and CKD [27,28]. In cultured podocytes, high glucose was shown to induce podocyte dedifferentiation through the up-regulation of miR-193a [29]. TGF-β1 was reported to up-regulate miR-192 in cultured glomerular mesangial cells and in glomeruli from streptozotocin-induced diabetic mice. The specific reduction of renal miR-192 by using a locked nucleic acid–modified inhibitor of miR-192 decreases renal fibrosis [30,31]. However, in cultured human proximal tubular cells (HK-2 cells), TGF-β1 led to a 51% reduction in miR-192 [26]. The miRs identified in these studies, including miR-192, miR-193a, miR-362-3p, miR-877-3p, miR-150-5p, and miR-15a-5p, could be informative but not necessarily have a role in the pathogenesis of human DKD. In our study, we examined the miR signature in nephrotic, biopsy-proven, isolated DN and found a completely different set of miRs. We demonstrated that miR-188-5p, miR-150-3p, miR-760, miR-3677-3p, miR-548ah-3p, miR-548p, miR-320e, and miR-23c were significantly up-regulated in nephrotic DN patients. A significant down-regulation of miR-133a-3p and miR-153-3p was also found in our study patients.

In a cell culture model, miR-188-5p is shown to regulate high glucose-induced epithelial-mesenchymal transition (EMT) in human proximal tubular cell lines through the PTEN/PI3K/Akt pathway [21]. By analyzing miRs from the patients’ urine exosomes, we demonstrated the pathologic role of miR-188-5p in nephrotic and biopsy-proven isolated DN. Notably, our analysis, based on KEGG and NIH DAVID Bioinformatics Resources, showed that the PI3K/Akt-signaling pathway appeared to play pathologic roles in isolated DN (Table 3). The circulating-exosomal miR-150-3p is reported to be related to albuminuria in patients with DN [22]. In line with this observation, our findings on up-regulated miR-150-3p revealed its pathologic role in isolated DN. Further experiments on the function of our newly-reported miRs are required to characterize their pathologic roles in isolated DN.

In addition to the PI3K-Akt-signaling pathway, we also showed that MAPK-signaling, human papillomavirus infection, Ras-signaling, endocytosis, and pathways regulating the pluripotency of stem cells could be the mechanisms underpinning DN. Some of these pathways have been reported in various experimental diabetes models. MAPK-signaling plays crucial roles in DN because it is related to inflammatory, oxidative, and apoptotic processes [32]; evidence of its activation has been demonstrated on the kidney biopsies of diabetic patients [33]. High glucose levels are known to induce Ras/Rac1-dependent superoxide formation in rat mesangial cells [34,35]. Although we were unable to specify the intra-renal location of our identified miRs, our functional analysis supported the findings derived from experimental diabetes and highlighted the pathologic roles of Ras-signaling in DN patients. Furthermore, the functional analysis of our identified miRs revealed a participation of the endocytosis pathway. This was of particular interest because it reflected the fact that all our study patients were nephrotic. In cell culture models, high glucose levels are known to reduce the megalin-mediated albumin endocytosis in renal proximal tubule cells [36]. Additionally, in renal proximal tubule cells, the inhibition of Akt is known to abrogate insulin-induced albumin endocytosis [37]. 

By using KEGG pathway prediction, we identified human papillomavirus infection and pathways regulating the pluripotency of stem cells as two novel pathways underpinning DN. In our results, AKT3, targeted by miR-320e (Table 4), served as the signaling hub linking the known and novel mechanisms of DN. Akt2 is known to protect podocytes in rodent diabetic models [38,39]. However, in humans, AKT3, not AKT2, is far more abundant in glomeruli than in tubules [40]. As we investigated pathologic miRs and pathways underpinning heavy proteinuria in DN, our findings on miR-320e and AKT3 were of particular importance.

Our study analyzed miRs from liquid (urine) biopsy specimens in clinical nephrotic, biopsy-proven DN patients. Compared to the previously-reported DN-related miRs, these newly-reported miRs could be the true regulators in the pathogenesis of DN. Functional analysis, based on our identified miRs, disclosed known and new pathways underpinning DN pathogenesis. These findings will inspire future research into the mechanisms underlying the pathogenesis of DN.

A major limitation of our study was the lack of real-time quantitative PCR-based validation of our identified miRs. However, unlike the known-reference miRs in cervical cancer and colorectal cancer [41,42], the reference miRs in DN remain unknown. An alternative approach to overcome this problem we would like to suggest is to test our reported deregulated miRs in various clinical and experimental models.

## 5. Conclusions

In conclusion, we reported a new urinary-exosomal miR signature in nephrotic, biopsy-proven DN patients. Their functional roles in DN pathogenesis were partly- supported by known pathways in previously-reported experimental diabetes models. Further experiments are required to investigate the functional roles of our newly-found target genes of each miR in the pathogenesis of DN.

## Figures and Tables

**Table 1:** Characteristics and clinical data of the study subjects.

Patient Number	1	2	3	4	5	6
Age (years)	54	38	46	29	60	58
Gender	Female	Male	Female	Female	Female	Female
Diabetes duration (years)	4	2	15	13	9	10
Diabetic retinopathy	(−)	(+)	(+)	(+)	(−)	(+)
Hypertension	(+)	(+)	(+)	(+)	(+)	(+)
eGFR (mL/min/1.73 m^2^) at biopsy	19	23	37	104	47	43
HbA1C (%) at biopsy	7.0	14.9	10.4	14.9	6.7	12.2
UPCR (mg/g) at biopsy	7662.2	11384.8	5470.6	11737.5	6617.3	20364.4
eGFR at 1 year after biopsy	13	8	23	52	30	32
HbA1C at 1 year after biopsy	8.2	6.6	7.3	10.0	6.9	7.4
Use of RAS blockade	(+)	(+)	(+)	(+)	(+)	(+)
Use of statin	(+)	(+)	(+)	(+)	(+)	(+)
Use of immunosuppressant	(+)	(−)	(−)	(−)	(−)	(−)

eGFR, estimated glomerular-filtration rate; HbA1C, glycated hemoglobin; UPCR, urinary protein-to-creatinine ratio; RAS, renin-angiotensin system.

**Table 2:** MicroRNAs (miRs) with the highest fold change in expression levels in nephroticdiabetic nephropathy (DN) patients.

miRNA	Fold Changes	Up/Down Regulation	*p*-Value	Reported Function or Altered Expression in Kidneys	References
miR-188-5p	15.8830569	Up	0.043876707	Regulates high glucose induced EMT in HK-2 cells via PTEN/PI3K/Akt pathway	[21]
miR-150-3p	14.9300289	Up	0.048010346	Upregulated circulating miRs in DN patients	[22]
miR-760	14.7636619	Up	0.022022315	N/A	
miR-3677-3p	14.6891608	Up	0.022071453	N/A	
miR-548ah-3p	13.8204449	Up	0.049986838	N/A	
miR-548p	13.8204449	Up	0.049986838	N/A	
miR-320e	13.6115628	Up	0.008895953	N/A	
miR-23c	13.4447561	Up	0.037456289	Inhibits pyroptosis in HK-2 cells	[23]
miR-133a-3p	-7.1177188	Down	0.03960008	Upregulated in OTA-intoxicated pig kidney	[24]
miR-153-3p	-9.8199843	Down	0.038408788	Abundant in class IV lupus nephritis	[25]

EMT, epithelial-mesenchymal transition; DN, diabetic nephropathy; OTA, ochratoxin A.

**Table 3:** Predicted pathways associated with the identified miRs in nephrotic DN patients.

ID	Description	GeneRatio	BgRatio	*p* Value	p.adjust	*q* Value
hsa04151	PI3K-Akt signaling pathway	65/847	354/7440	0.000049644	0.000533552	0.000363517
hsa04010	MAPK signaling pathway	61/847	295/7440	0.000001816	0.000070128	0.000047779
hsa05165	Human papillomavirus infection	54/847	339/7440	0.006064587	0.024417472	0.016635988
hsa04014	Ras signaling pathway	53/847	232/7440	0.000000358	0.000026492	0.000018049
hsa04144	Endocytosis	50/847	244/7440	0.000020338	0.000392778	0.000267606
hsa04550	Signaling pathways regulating pluripotency of stem cells	33/847	139/7440	0.000025650	0.000403281	0.000274761

**Table 4:** Target genes, functional pathways and the miRs in nephrotic DN patients.

PI3K-Akt Signaling Pathway		MAPK Signaling Pathway		Human Papillomavirus Infection	
Target Genes	miRs	Target Genes	miRs	Target Genes	miRs
EIF4E	miR-548p	MAP3K7	miR-548p	FZD3	miR-23c
FGFR1	miR-548p	FGFR1	miR-548p		
PRLR	miR-548p, miR-23c	MKNK2	miR-548ah-3p	HDAC2	miR-548p, miR-548ah-3p
CCND1	miR-548ah-3p	TGFBR1	miR-548ah-3p	CCND1	miR-548ah-3p
PRKAA2	miR-548ah-3p	RPS6KA3	miR-548ah-3p	ATP6V0A2	miR-548ah-3p
PPP2R5E	miR-548ah-3p	NF1	miR-548ah-3p	PPP2R5E	miR-548ah-3p
AKT3	miR-320e	AKT3	miR-320e	AKT3	miR-320e
CDK6	miR-320e	STK4	miR-320e	CDK6	miR-320e
FGF1	miR-760	FGF1	miR-760		
PDPK1	miR-760				
**Ras Signaling Pathway**		**Endocytosis Pathway**		**Signaling Pathways Regulating Pluripotency of Stem Cells**	
**Target Genes**	**miRs**	**Target Genes**	**miRs**	**Target Genes**	**miRs**
FGFR1	miR-548p	DNM3	miR-548p	FGFR1	miR-548p
NF1	miR-548ah-3p	TGFBR1	miR-548ah-3p	IL6ST	miR-188-5p, miR-320e
AKT3	miR-320e	CHMP1B	miR-548ah-3p	AKT3	miR-320e
STK4	miR-320e	NEDD4L	miR-23c	ZFHX3	miR-548ah-3p
KSR2	miR-320e, miR-760	CBL	miR-760	SKIL	miR-548ah-3p
FGF1	miR-760	SNX1	miR-760	FZD3	miR-548ah-3p, miR-23c
				SMAD4	miR-760

Background color is simply for better visualization.
